# Mapping Forensic Psychiatry Education Across Europe: Insight from EFPT members

**DOI:** 10.1192/j.eurpsy.2025.399

**Published:** 2025-08-26

**Authors:** F. M. Monshizadeh Tehrani, B. Akan, B. Özel, S. Kukurt, E. Ilgin, V. H. Santos, M. Nouri, D. Zani, K. Krasteva, P. Viktorova, M. Godschalk, G. Autissier, A. Compaired, F. Mustač, L. Sanduleac, O. Nikolaieva, D. A. Zaharie, J. Halili, A. Avalishvili, G. Longo, N. De Ridder, A. Braicu, S. Shokatpour

**Affiliations:** 1 Forensic Psychiatry Working Group, European Federation of Psychiatric Trainees, Brussels, Belgium; 2 Zanjan University of Medical Science, Zanjan; 3 Faculty of Psychology and Educational Sciences, Islamic Azad University, Islamshahr Branch, Tehran, Iran, Islamic Republic Of

## Abstract

**Introduction:**

Forensic psychiatry transcends legal and cultural boundaries across Europe, but specialization and training remain inconsistent. With freedom of movement in most European countries, psychiatrists accredited in one country can practice in others if they meet language requirements. Therefore, harmonizing psychiatric education and practice is crucial and aligns with the European Federation of Psychiatry Trainees (EFPT)’s goals.

**Objectives:**

This study aims to map the current state of forensic psychiatry education across Europe, focusing on its recognition as a specialty on its own or subspecialty, training structure, and financial implications. It also assesses whether general adult psychiatry (GAP) and child and adolescent psychiatry (CAP) trainees receive adequate forensic psychiatry education, identifying gaps and variations across countries.

**Methods:**

Data was collected via an online survey distributed to European National Trainee Association (NTA) representatives in the EFPT through Google Forms in August 2024. Responses from non-European countries and incomplete entries were excluded. The final dataset was analyzed using SPSS 24.

**Results:**

A total of 29 participants, including 24 GAP trainees (82.8%), 2 CAP trainees (6.9%), and 3 specialists (10.3%), from 20 European countries responded to the survey. Forensic psychiatry was recognized either as a specialty or subspecialty in 13 counctries (65%) with 20 (69%) of participants confirming its recognition. 38% reported forensic training lasts less than 1 year or lacks a formal program. Financial support varied as well, with some countries offering full subsidies, while others required trainees to cover costs. Forensic psychiatry was included in the training of 66.7% of GAP trainee and 50% of CAP trainees, though the depth of exposure differed. Notable gaps were found particularly in risk assessment, expert witness training, and competency evaluations.

**Conclusions:**

This study reveals significant variation in the recognition, structure, and delivery of forensic psychiatry training across Europe. While some countries offer well-defined programs, discrepancies in accessibility and comprehensiveness persist. These findings highlight the need for standardized curricula to ensure consistent training. Enhancing forensic psychiatry education is crucial for preparing future psychiatrists and ensuring high-quality psychiatric contributions in legal contexts and harmonization of forensic training across Europe.

**Disclosure of Interest:**

None Declared

